# Case of Extrophy of the Bladder

**Published:** 1855-08

**Authors:** John Bartlett

**Affiliations:** Louisville


### ART. III.—CASE OF EXTROPHY OF THE BLADDER.

BY JOHN BARTLETT, M. D.

Andrew Miller, aged twenty, was born at Frostburgh, Md. His father and mother were well formed, and Andrew is the youngest of four well formed children, he is five feet nine inches in height, of large frame, but not muscular. Immediately above the site of the pubic bones is the protruded posterior wall of the bladder, forming an irregular tumor which measures in its transverse diameter, two and a half inches; in its vertical, one and one fourth inches. Ordinarily the projection of the tumour beyond the skin is as much as an inch, and he has not observed that its prominence depended upon the distention or flaccidity of his abdominal organs. But after being recumbent for some hours the tumor disappear and there is a concavity in its stead. The surrounding skin has the appearance of having encroached upon the mucous membrane of the tumour, which is very vascular. Immediately below the tumour is the imperfect penis, two inches in length, with a transverse diameter of one and five-eighths inches. Its upper surface is covered with a mucous membrane throughout, except one eight of an inch on each side, which is supplied with skin. Here the form of the corpora cavernosa is distinctly seen, and on this surface toward the right side, and at the junction of the posterior third with the anterior two-thirds of the penis a small slit is observable. It is supposed to be the orifice of one of the ejeculatory ducts. The penis has an imperfect head, flattened from above downwards ; at its apex a depression corresponding to the site of the urethral orifice is seen. The lower surface of the penis is covered with skin which is loosely attached to the deeper structures, and wrinkled laterally. It forms a prepuce redundant on one side. The scrotum and testicles are perfect, though the testicles are smaller than usual. There is an absence of the bodies of the pubic bones, the interval between the horizontal rami measuring three and a quarter inches.

The termination of the left ureter is seen one fourth of an inch internal to the left extremity of the tumour and at its inferior margin it presents a cup-shaped cavity admitting the end of a silver probe. The termination of the right ureter is not easily discernable, but by forcibly raising the tumour and depressing the penis, its orifice was discovered as an almost imperceptible slit, at the junction of the tumour and penis, five-eighths of an inch internal to the right extremity of the tumour, and one inch and five-eighths from its fellow.

Three-quarters of an inch above the tumour is a slight, smooth, circular elevation of the skin, three-fourths of an inch in diameter, it is the umbilicus.

The parts are plentifully supplied with hair, which is matted with a white deposit, probably phosphate of lime.

From the orifice of the left ureter escapes, at intervals of a minute or two, a large drop or a small jet of urine, which running between the tumour and the penis mingles with the supply from the right ureter and flows over the side of the penis.

He catches the urine in cloths which are frequently changed; there is a greater flow of it in winter than in summer, and, in winter he suffers much from excoriation, being sometimes for weeks unable to walk; in summer he is free from this annoyance. He refrains from tea and coffee, because they increase the urinary deposit, and the odor of putrescent urine which is offensive to others and an affliction to himself.

The tumour is very sensitive. He can walk pretty well by guarding it from friction. It is very sensitive to cold, so that he suffers extremely with it in the winter if he be exposed. His voice and manners are effeminate. He has "the same passion for women as other men," and several times a week he has a seminal emission which is attended with pleasurable sensations. The semen, he says, wells up from between the tumor and the penis, and comes from both sides.

When questioned as to the probable cause of this deformity nothing is revealed save that his parents at the time of his birth lived very unpleasantly together.

*Louisville*, *Aug.*, 16, 1855.

